# Shortlasting, Unilateral, Neuralgiform, Headache Attacks with Conjunctival Injection, Tearing, Sweating and Rhinorrhea: The Term and New View Points

**DOI:** 10.3389/fneur.2018.00262

**Published:** 2018-04-23

**Authors:** Fabio Antonaci, Torbjørn Fredriksen, Juan A. Pareja, Ottar Sjaastad

**Affiliations:** ^1^Department of Brain and Behavioral Sciences, C. Mondino National Institute of Neurology Foundation, IRCCS, University of Pavia, Pavia, Italy; ^2^Department of Neurosurgery, St. Olavs Hospital, Trondheim University Hospitals, Trondheim, Norway; ^3^Department of Neurology, University Hospital Fundación Alcorcón, Madrid, Spain; ^4^Department of Neurology, St. Olavs Hospital, Trondheim University Hospitals, Norwegian University of Science and Technology, Trondheim, Norway

**Keywords:** shortlasting unilateral neuralgiform conjunctival injection and tearing, short-lasting unilateral neuralgiform headache attacks with cranial autonomic symptoms, trigeminal neuralgia, autonomic nervous system dysfunction, unilateral headaches

## Abstract

A solitary patient with symptoms similar to those of shortlasting unilateral neuralgiform conjunctival injection and tearing Short lasting, Unilateral, Neuralgiform headache attacks with Conjunctival injection, Tearing, sweating and rhinorrhoea (SUNCT) was first mentioned in 1978. The term SUNCT was first used in 1991. SUNCT is an acronym; the “S” signifies “Shortlasting”; the “U” symbolizes “Unilateral”; “N” stands for “Neuralgiform”; the “C” for “Conjunctival injection”; and “T” for “Tearing.” The term short-lasting unilateral neuralgiform headache attacks with cranial autonomic symptoms were marketed in 2004. The terminology and new view points are discussed and nosography proposal for SUNCT is presented.

## Background

This overview deals only with the pure clinical approach to shortlasting unilateral neuralgiform conjunctival injection and tearing Short lasting, Unilateral, Neuralgiform headache attacks with Conjunctival injection, Tearing, sweating and rhinorrhoea (SUNCT) diagnosis. The work-up, i.e., identification of neurovascular compression is another story.

A solitary patient with symptoms similar to those of SUNCT was first mentioned in 1978 (1). This case obviously caused nosological problems—for the authors. When three cases were known to the authors—worldwide—in 1989, this headache was described in detail, under the heading: “Shortlasting, unilateral neuralgiform headache attacks with conjunctival injection, tearing, sweating, and rhinorrhea” (2). The term SUNCT was first used in 1991 (3). SUNCT is an acronym; the the “S” signifies “Shortlasting”; the “U” symbolizes “Unilateral”; “N” stands for “Neuralgiform”; the “C” for “Conjunctival injection”; and “T” for “Tearing.” Thus, the heading/title (from 1989) is more extensive than the term SUNCT (from 1991), in that there in the first one are *four* autonomic variables and not only *two*. Many workers in the field have not realized that sweating and rhinorrhea also belong to the full term. Even in the main introduction of SUNCT in the IHS 2004 classification (3.3; p. 46) (4) it is only mentioned “Conjunctival injection and Tearing”—and not forehead sweating and rhinorrhea. The same is the case in the description in the 2013 edition (3.3.1; p. 668.) (5). The reason why the acronym was cut off at “tearing” was mainly that the acronym should be as short and succinct as possible. That being said, already with only three patients, it was felt that conjunctival injection and tearing probably were the two most spectacular autonomic abnormalities in SUNCT, with a regularity and a vehemence, unlike those of other autonomic abnormalities (6–8). For those reasons, they ought to be treated differently than other autonomic abnormalities, in SUNCT descriptions. It is a breath-taking experience to watch an attack of SUNCT with a scene shift within a few seconds, and the two most amazing phenomena are conjunctival injection and lacrimation. SUNCT is the most distinct of the headaches whose birth we have witnessed. It should, however, be emphasized that we never have felt that conjunctival injection and tearing should be *obligatory* phenomena in SUNCT. It is probably possible to make the diagnosis of SUNCT in the absence of conjunctival injection/lacrimation. The position of forehead sweating, symptomatic side, is a little more problematic: it seems invariably or close—to—invariably to be augmented during paroxysm (6). However, this type of sweating is mostly subclinical. Clearly, the extent of attack-related forehead sweating is less that in, e.g., cluster headache. Generally, sophisticated methods are required to detect it. It is unlikely that measurement of sweating will be part of routine headache practice in the foreseeable future. Also for that reason, forehead sweating was not made part of the acronym.

Already the first review on SUNCT from 1997, with 21 cases (7) showed that conjunctival injection and tearing, *de facto*, were in a class of their own, being present invariably/close—to—invariably during attack. Also rhinorrhea was present frequently, i.e., in ca. 2/3 of the cases (7). Forehead sweating had probably not been properly *measured* in that context; no emphasis can, therefore, be placed upon the figures given. We have set no limit to inclusions of autonomic variables in SUNCT. Such inclusions must be based on observations, evidence, and logic, and not on mere speculations. We have, in the term SUNCT, as well as in the heading of our 1989 article (2) proposed a probable succession as to prevalence and importance of autonomic variables.

## The IHS Criteria

Then, the IHS 2004 version (4) and later the 2013 version (5) of the headache classification provided instructions that made conjunctival injection and tearing obligatory phenomena, in SUNCT. To introduce a “must” in this connection is, as we see it, a misunderstanding. That conjunctival injection and tearing are included in the acronym does not imply that these are the only autonomic variables, in SUNCT, and that their presence is absolute. Beside “sweating and rhinorrhoea,” legion autonomic disturbances can be incorporated. Thus, in the early review (7), there were around 10 more autonomic variables, and one or more of them that might be valid. There is considerable uncertainty concerning duration of attacks. Duration has been given as (4): 5–240 s (and for SUNA: 2 s−10 min). In the IHS 2013 edition (5), duration was stipulated as: 1-s, probably for both SUNCT and SUNA. There may accordingly be reasons to be confused. We propose a duration of 10–120 s; (or: rather, 10–90 s), based upon the exact timing of 348 attacks (8). Few attacks lasted <10 s and only 4.3% >120 s. With an upper border of 90 s, the border will be exceeded by only ca. 9% of the attacks, a number that is allowable in biology. Accordingly, with such a design, one will have to allow that the borders occasionally are exceeded.

## Short-Lasting Unilateral Neuralgiform Headache Attacks with Cranial Autonomic Symptoms

Then, the term SUNA was marketed in 2004 (4, 9).

One can get the impression that there has been some hard striving to find arguments to justify that term (4). One apparently finally came up with three arguments in favor of SUNA. The first one was the “notable problems” with SUNCT that conjunctival injection and tearing was a “must” (a proposition set forth by the actual committee, as far as the present authors know; at least not by us). The irrational nature of this claim has been dealt with in detail in the foregoing.

Second, the pain of SUNCT paroxysms may be difficult to distinguish from that of first branch trigeminal neuralgia. That may be so. However, SUNCT is not identical to (I) branch trigeminal neuralgia. In (I) branch trigeminal neuralgia, pain may—over time—spread to branches (II). And (III), while the pain of SUNCT tends to be more stationary. Furthermore, the various pain characteristics differ in the two disorders: (1) Pain quality: neuralgia pain is generally sharper and more intense. A conversation can frequently be carried out during a SUNCT paroxysm. (2) A neuralgia attack tends to be shorter than a SUNCT paroxysm (10). (3) The frequency of neuralgia paroxysm, at the top of the curve, tends to be higher than that of SUNCT. (4) Paroxysms are frequently triggered in both types of attacks, but more regularly in the neuralgia than in SUNCT (2, 10). In neuralgia, just a minimal touch or a puff of wind may suffice, whereas in SUNCT, a slightly “rougher” stimulus may be necessary, e.g., rubbing of the skin (7). (5) In neuralgia, there typically is a post-ictal refractory period, whereas in SUNCT, such period may or may not be present, usually not (7). (6) Nocturnal attacks are frequent in neuralgia, but are rarer in SUNCT. Pareja et al. registered 585 attacks (8), and only 1–2% of them appeared during night, depending upon how night was defined. This is actually one of the best differential diagnostic clues available. (7) Another decisive difference is that carbamazepine has a more marked effect in the neuralgia (11) than in SUNCT.

On top of that, the autonomic system involvement differs vastly between the neuralgia and SUNCT, both quantitatively and qualitatively. Only 11% of the first branch neuralgia patients have the combination of conjunctival injection, lacrimation, and rhinorrhea (10), whereas ca. 2/3 of SUNCT patients exhibit this combination. If, in a comparison of the two types of paroxysms, the value 1 is given for the mere presence of each of the variables: conjunctival injection, tearing, and rhinorrhea, the mean value in the neuralgia group was 0.68 (out of a maximum of 3.0), versus a mean of 2.63 in SUNCT (*p* < 0.0000005, chi-square test) (10). Rhinorrhea seemed to be the solitary autonomic variable that best separates the neuralgia and SUNCT (*p* < 0.0006). There is thus little doubt that in the neuralgia and SUNCT, the three major autonomic variables behave differently. This difference can probably partly, but not entirely, be explained by the usually shorter duration of paroxysms in the neuralgia. The topic of differential diagnostic problems between the neuralgia and SUNCT is to a large extent an artificial one. And this problem in no way justifies the creation of this group, SUNA. In this context, the very presence of autonomic disturbances has been focused and not the grade of the abnormality. It should be pointed out that there is no single test that can identify SUNCT with 100% certainty.

All in all, the clinical distinction between the neuralgia and SUNCT should generally not be a problem.

The third counterargument against SUNCT was that the description of attack frequency was “rather unhelpful,” given “the breadth of variation it allows.” The frequency “allowed” by the IHS was 3–200 attacks per day (4). In SUNA, it was proposed to be: >1 day (“for more than half of the time,” whatever that is supposed to mean).

The situation is as follows: we have never been asked by the committee about such frequency. The aforementioned figures probably stem from the actual committee itself. The standard has been set, by the committee, and later it is argued against what they themselves have proposed. The frequency of attacks in SUNCT is actually rather well known (8). The frequency of SUNCT paroxysm does not *per se* represent an argument against the purity of SUNCT as such. It remains an enigma how the introduction of SUNA, with its frequency of attacks, could aid in a situation of diagnostic ambiguity in SUNCT. One may rather have a hunch that one is faced with a case of ulterior motive.

## New Information on SUNCT

A review article on the SUNCT syndrome (11) throws some new light upon SUNCT and its relationship to SUNA. This review covers the period from ca. 2000 to 2012. The first review on SUNCT, comprising 21 cases, covered the period 1978–1997, when much of the foundation for SUNCT was laid (7). Patients from the first review are for some unknown reason not included in the second review. Some major points from the last review will be dealt with in the present communication.

### Gender

In this review, there were 109 males and 74 females with SUNCT, with a male/female ratio of: 109:74 = 1.47. In SUNA, there were 11 males and 19 females, with a sex ratio of: 11:19 = 0.58. The two sex ratios differ significantly, i.e., *p* = 0.01, with a binominal model. This is a serious finding. The chances that SUNCT and SUNA represent the same type of patient population are limited.

### SUNCT and SUNA: Total Number of Patients

The total number of SUNCT patients amounted to: males: 109; females: 74 = 183. The total number of SUNA patients was: males: 11; females: 19 = 30. There were accordingly 6.1 times as many SUNCT as SUNA patients. With the present diagnostic system, SUNA seems to be a tiny group, or perhaps a conglomerate of ill-defined, minute groups. The authors behind the original description of SUNA foresaw (4) that SUNA would be a big group that would engulf SUNCT.

## Autonomic Disturbances

The occurrence of conjunctival injection and tearing during attacks (11) is significantly higher in SUNCT than in SUNA (*p* < 0.001 for both, chi-square test). Conjunctival injection and tearing were both present in 100% of the SUNCT cases. These figures indicate that SUNCT and SUNA are alien to each other. Also rhinorrhea differed clearly, but not significantly between SUNCT and SUNA (*p* = 0.06).

One particular feature is “sensation of fullness in the ear” (symptomatic side); this appears on a list of seven different, autonomic factors (3.3.C: 2013 version) (5), on line with, e.g., conjunctival injection and/or tearing. Each one of these seven factors would in principle satisfy the obligation of autonomic involvement in SUNA-see 3.3. The weight of the two solitary factors in 3.3.C.1, i.e., conjunctival injection and lacrimation, is closer defined: in SUNCT (see 3.3.1.B), both must be present, whereas in SUNA (3.3.2.B), “only one or neither of conjunctival injection or lacrimation” are to be present. In other words, SUNA diagnosis can be (rather: should be) made without any of the two central, autonomic variables, or with one of them present. Conjunctival injection and lacrimation almost invariably co-exist. It will, therefore, in clinical practice close—to—never happen that one of these two autonomic features appears alone; this should probably also concern attacks of SUNA (It should be emphasized that the intensity of the various autonomic phenomena is not taken into consideration in this classification), In that case, the 3.3.2.B, concerning SUNA, should accordingly probably be changed, for example, to: “Neither conjunctival injection nor lacrimation.” Obviously, the consequences will be grave: with almost surgical precision, the link between SUNCT and SUNA will be cut. It will be impossible to both have and not have the combination of conjunctival injection and lacrimation in one and the same patient. It will accordingly be close to impossible to establish the co-existence of SUNCT and SUNA in one solitary patient. In the last review (11), such a co-existence was nevertheless diagnosed in some patients. Cases of solitary SUNA are also not infrequently misdiagnosed.

The observation that a patient with full-blown attacks of SUNCT, in a given phase, may have mild attacks, inclusive of weak/hardly noticeable autonomic signs, concerning also conjunctival injection and tearing, does not indicate that the patient, in addition, has got SUNA. It is extremely important to realize this.

A problem is that SUNA hardly can be diagnosed on the basis of “ear fullness” alone, in spite of what is stated in 3.3.2. and 3.3.C. This symptom does not seem to be specific enough. Such fullness pops up in the diagnostic criteria for both cluster headache, CPH, and hemicrania continua (5). Moreover, its presence is apparently not mentioned in a single case of SUNCT, neither in the most recent review (11), nor in the previous review (7). This criterion does, therefore, not seem to defend the position allotted to it in the present diagnostic system for SUNA.

## Conclusion

Shortlasting unilateral neuralgiform conjunctival injection and tearing is a headache taken out of real life; it is a headache based on sound principles. SUNA has been supposed to belong to the SUNCT cycle. Unfortunately, this headache seems to be based on fragile constituents, if the main intention was to keep the bonds to SUNCT intact. It is stated (5) that in SUNA there should be “—only one or neither of conjunctival injection and lacrimation—.” In demanding this, the bonds to SUNCT are more or less cut. It is well established that conjunctival injection and lacrimation have a disposition to *coexist* in SUNCT. Such coexistence is not to be present in SUNA (5).

Moreover, attacks of SUNA could last up to 10 min (4). The “S” of SUNCT stands for short lasting. One can hardly characterize 10 min as being short lasting. The invention of SUNA reminds one of writing desk medicine/neurology. When one starts tampering with the core properties of a headache syndrome, one must be prepared for immense obstacles.

Shortlasting unilateral neuralgiform conjunctival injection and tearing comes out of this test (11) more or less as expected. The bonds between SUNA and SUNCT have more or less been cut. SUNCT is a headache in its own right. Due to the different attitude to the conjunctival injection/tearing combination in SUNCT and SUNA it becomes close-to-impossible to diagnose both SUNCT and SUNA in the same individual. The foregoing can prove to be one of the most important outcomes of the present article. The future of SUNA may seem gloomy.

Due to the various uncertainties that have been brought to light in the foregoing, it is felt that an upgrading of the criteria would be appropriate.

## SUNCT: Proposed Diagnostic Criteria

### Description

SUNCT is an acronym for: Short lasting, Unilateral, Neuralgiform headache attacks with Conjunctival injection, Tearing, sweating and rhinorrhoea (3).

SUNCT is a short lasting, non-pulsating, unilateral headache of moderate to severe intensity, the maximal pain mainly being in the periocular area and forehead. The paroxysms coexist with ipsilateral autonomic phenomena, such as conjunctival injection, tearing, rhinorrhea, and sweating, the latter generally being subclinical. Generally, the attack frequency is high—with multiple attacks per 24 h—but highly varying, and with a tendency to clustering, and with largely diurnal attacks. Paroxysms may frequently be precipitated from sensitive trigger zones.

### Diagnostic Criteria

- Unilateral headache
- The moderate to intense, non-pulsating pain has its maximum in periocular area/forehead
- Attack precipitation from particular trigeminal/extratrigeminal zones
- Duration, paroxysms; generally: 10–90 s. These borders may occasionally be exceeded
- Frequency: 6–77/day (range^1^) These borders may be exceeded
- Autonomic symptoms and signs, accompanying the attack.
  (A) Invariably, or close-to-invariably present
    1. Conjunctival injection
    2. Tearing
    3. Slight, visible over-ventilation during attacks
  (B) Less frequently present, but still rather frequent autonomic phenomena
    4. Rhinorrhea^2^
    5. Nasal obstruction (see text footnote 2)
    6. Forehead sweating.^3^
  (C) Other autonomic disturbances
    1. Eyelid edema, also outside paroxysms
    2. Erythema; eyelids, face
    3. Telangiectasia, mainly eyelids: permanent, marked during attack
    4. Eyelid vasodilatation, most marked during attack.

## Author Contributions

FA: ref search and paper reviewing, TF: writing and reviewing the paper, JP: ref search and paper reviewing, and OS: study design, writing and reviewing the paper. All authors read and approved the final manuscript.

## Conflict of Interest Statement

The authors declare that the research was conducted in the absence of any commercial or financial relationships that could be construed as a potential conflict of interest.

## Footnotes

^1^Figures according to Ref. (8).

^2^The “transport route” from the production site to the nares is rather long for the produced phlegm. If the paroxysm is rather short, the mucous material may not have reached the nares at the end of the attack. Its presence in the nostril may be verified, by light, cotton, etc. In scientific publications, it should be specified whether the presence of the phlegm has been established directly or indirectly.

^3^Forehead sweating is close to invariably present with laboratory methods. It is, however, more often than not “subclinical.”

## Abbreviations

SUNCT, shortlasting unilateral neuralgiform conjunctival injection and tearing; SUNA, short-lasting unilateral neuralgiform headache attacks with cranial autonomic symptoms.
