# Corrigendum: Shortlasting, Unilateral, Neuralgiform, Headache Attacks With Conjunctival Injection, Tearing, Sweating and Rhinorrhea: The Term and New View Points

**DOI:** 10.3389/fneur.2018.00547

**Published:** 2018-07-18

**Authors:** Fabio Antonaci, Torbjørn Fredriksen, Juan A. Pareja, Ottar Sjaastad

**Affiliations:** ^1^Department of Brain and Behavioral Sciences, C. Mondino National Institute of Neurology Foundation, IRCCS, University of Pavia, Pavia, Italy; ^2^Department of Neurosurgery, St. Olavs Hospital, Trondheim University Hospitals, Trondheim, Norway; ^3^Department of Neurology, University Hospital Fundación Alcorcón, Madrid, Spain; ^4^Department of Neurology, St. Olavs Hospital, Trondheim University Hospitals, Norwegian University of Science and Technology, Trondheim, Norway

**Keywords:** shortlasting unilateral neuralgiform conjunctival injection and tearing, short-lasting unilateral neuralgiform headache attacks with cranial autonomic symptoms, trigeminal neuralgia, autonomic nervous system dysfunction, unilateral headaches

The title of the original article was changed from *Shortlasting Unilateral Neuralgiform Conjunctival Injection and Tearing Syndrome: The Term and New View Points* to *Shortlasting, Unilateral, Neuralgiform, Headache Attacks With Conjunctival Injection, Tearing, Sweating and Rhinorrhea: The Term and New View Points*.

In addition, a correction has been made to the last sentence of the section *The IHS Criteria* and should read:

“Duration has been given as [4]: 5–240 s (and for SUNA: 2 s – 10 min).”

The corrected paragraph appears below:

## The IHS criteria

Then, the IHS 2004 version (4) and later the 2013 version (5) of the headache classification provided instructions that made conjunctival injection and tearing obligatory phenomena, in SUNCT. To introduce a “must” in this connection is, as we see it, a misunderstanding. That conjunctival injection and tearing are included in the acronym does not imply that these are the only autonomic variables, in SUNCT, and that their presence is absolute. Beside “sweating and rhinorrhoea,” legion autonomic disturbances can be incorporated. Thus, in the early review [7], there were around 10 more autonomic variables, and one or more of them that might be valid. There is considerable uncertainty concerning duration of attacks. Duration has been given as [4]: 5–240 s (and for SUNA: 2 s-10min).

The authors apologize for this error and state that this does not change the scientific conclusions of the article in any way.

The original article has been updated.

### Conflict of interest statement

The authors declare that the research was conducted in the absence of any commercial or financial relationships that could be construed as a potential conflict of interest.

